# Early life stress, prenatal secondhand smoke exposure, and the development of internalizing symptoms across childhood

**DOI:** 10.1186/s12940-023-01012-8

**Published:** 2023-08-25

**Authors:** Mariah DeSerisy, Jacob W. Cohen, Jordan D. Dworkin, Jeanette A. Stingone, Bruce Ramphal, Julie B. Herbstman, David Pagliaccio, Amy E. Margolis

**Affiliations:** 1 Department of Epidemiology, Mailman School of Public Health, Columbia University, 722 West 168th Street, New York, NY 10032 USA; 2 Department of Psychiatry, Vagelos College of Physicians and Surgeons, Columbia University, 1051 Riverside Drive, New York, NY 10032 USA; 3 Division of Child and Adolescent Psychiatry, New York State Psychiatric Institute, 1051 Riverside Drive, New York, NY 10032 USA; 4 Harvard Medical School, 25 Shattuck Street, Boston, MA 02115 USA; 5 Department of Environmental Health Sciences, Mailman School of Public Health, Columbia University, 722 West 168th Street, New York, NY 10032 USA; 6 Columbia Center for Children’s Environmental Health, Mailman School of Public Health, Columbia University, 722 West 168th Street, New York, NY 10032 USA

**Keywords:** Secondhand Tobacco smoke, Early life stress, Internalizing problems, Child Development

## Abstract

**Background:**

Prior findings relating secondhand tobacco smoke (SHS) exposure and internalizing problems, characterized by heightened anxiety and depression symptoms, have been equivocal; effects of SHS on neurodevelopment may depend on the presence of other neurotoxicants. Early life stress (ELS) is a known risk factor for internalizing symptoms and is also often concurrent with SHS exposure. To date the interactive effects of ELS and SHS on children’s internalizing symptoms are unknown. We hypothesize that children with higher exposure to both prenatal SHS and ELS will have the most internalizing symptoms during the preschool period and the slowest reductions in symptoms over time.

**Methods:**

The present study leveraged a prospective, longitudinal birth cohort of 564 Black and Latinx mothers and their children, recruited between 1998 and 2006. Cotinine extracted from cord and maternal blood at birth served as a biomarker of prenatal SHS exposure. Parent-reported Child Behavior Checklist (CBCL) scores were examined at four timepoints between preschool and eleven years-old. ELS exposure was measured as a composite of six domains of maternal stress reported at child age five. Latent growth models examined associations between SHS, ELS, and their interaction term with trajectories of children’s internalizing symptoms. In follow-up analyses, weighted quintile sum regression examined contributions of components of the ELS mixture to children’s internalizing symptoms at each time point.

**Results:**

ELS interacted with SHS exposure such that higher levels of ELS and SHS exposure were associated with more internalizing symptoms during the preschool period (β = 0.14, *p* = 0.03). The interaction between ELS and SHS was also associated with a less negative rate of change in internalizing symptoms over time (β=-0.02, *p* = 0.01). Weighted quintile sum regression revealed significant contributions of maternal demoralization and other components of the stress mixture to children’s internalizing problems at each age point (e.g., age 11 WQS β = 0.26, *p* < 0.01).

**Conclusions:**

Our results suggest that prior inconsistencies in studies of SHS on behavior may derive from unmeasured factors that also influence behavior and co-occur with exposure, specifically maternal stress during children’s early life. Findings point to modifiable targets for personalized prevention.

**Supplementary Information:**

The online version contains supplementary material available at 10.1186/s12940-023-01012-8.

## Background

Although the last 50 years has seen tremendous progress towards reducing smoking among American adults [1], 13% remain active smokers [2]. Further, 11 states have no public smoking laws [3], leaving roughly 58 million non-smoking Americans exposed to secondhand, or environmental, tobacco smoke (SHS; [4]). As a result of structural racism and environmental injustice, relative to White women, Black women are disproportionately exposed to SHS [5] leading to disproportionate exposure during pregnancy. Specifically, in the workplace and public settings, Black Americans are disproportionately exposed to SHS compared to their white counterparts [6]; in private settings, such as homes and vehicles, Black individuals are also exposed to SHS at higher rates than white individuals [7, 8]. Such prenatal SHS exposure has wide reaching impacts on children’s later behavior and health [1, 9].

Equivocal evidence links prenatal SHS with internalizing symptoms. Some studies report strong associations between prenatal SHS and child internalizing problems, characterized by heightened anxiety and depression symptoms [10–12]; however, other studies do not [13–16]. Recent advances in environmental epidemiology highlight the importance of examining the combined effects of multiple chemical or social exposures that act as neurotoxicants, or co-exposures [17], given that people are rarely exposed to one neurotoxicant at a time [18]. Early life stress (ELS), consisting of parental stressors and adversities shared by both parents and children, have been shown to act as neurotoxicants [19–21]. Parental adversity and psychological distress are among the most robust and well-replicated risk factors for child psychopathology and mental health symptoms [22–24]. The mechanisms driving associations between maternal stress and child mental health symptoms are diverse and intersecting. For example, maternal stress can influence parenting style [25–27], early attachment and bonding [28, 29], and family processes, such as stress, cooperative caregiving, and home environment [30–32]. Intergenerational transmission of mental health symptoms may also be biological, with emerging evidence suggesting genetic, epigenetic, and physiological (e.g., oxytocin, immune, etc.) mechanisms underlying offspring vulnerability to psychiatric disorders [33–35]. Together, these findings highlight the importance of understanding the influence of maternal stress and shared adversities on children’s internalizing symptoms. In order to clarify prior conflicting findings in studies examining associations between ETS exposure and children’s internalizing symptoms, we examine if maternal stress during children’s early life compounds or is compounded by *prenatal* SHS exposure.

Given that maternal stress during children’s early life is a known risk factor for internalizing symptoms [35], we propose that it may serve as a critical effect modifier in associations between SHS and internalizing symptoms. Moreover, SHS and stress commonly co-occur [36], which can be understood theoretically through the stress process perspective [37] and empirically through the high rates of co-occurrence of parental stressors, nicotine dependence, and child maltreatment [36, 38, 39]. Shared and cascading effects of SHS and parental adversity (e.g., parental perceived stress, psychological distress, economic hardship, intimate partner violence, maternal demoralization, neighborhood quality, and lack of social support) on children’s wellbeing may also operate through shared neurobiological targets or synergistic effects on brain function [40, 41]. For example, animal models document that prenatal nicotine exposure combined with stress associated with maternal separation from pups during early infancy causes increased time spent immobile during a forced swim test, reflecting depressive-like behavior and anhedonia [42]. These exposures were also associated with an increased number of neurons in the amygdala and a decreased number of neurons in the ventral tegmental area, pointing to a possible neural mechanism by which co-exposure results in greater internalizing behaviors [42]. Although the effects of combined exposure to SHS and stress on mental health in humans have not yet been examined, their interaction has been linked with increased cognitive problems [43]. In addition to static effects on behavior, exposures may influence trajectories of development. Prior findings indicate relative stability or decreases in internalizing symptoms amongst community samples of youth before age 12 and then sharp increases into adolescence, particularly among girls [44–47]. Notably, these trajectories are shaped by stress, with maternal stress and psychopathology strongly associated with elevations in internalizing symptoms [44, 46–48]. Critically, studies reporting effects of prenatal SHS on internalizing symptoms have not examined longitudinal change in these symptoms over time [10–12]. How the combined effects of SHS and maternal stress during children’s early life may influence the trajectory of internalizing symptoms remains an important and understudied area.

The current study sought to address these gaps in knowledge by examining how combined exposure to prenatal SHS and maternal stress during children’s early life increases internalizing symptoms and influences development in a prospective birth cohort of non-smoking Black and Latinx mothers and their children (N = 482). Given prior findings from animal models and human epidemiologic studies, we hypothesized that prenatal exposure to SHS combined with maternal stress during children’s early life would result in elevated internalizing symptoms in youth that would remain present over time.

## Methods

### Participants

**Fig. 1:**
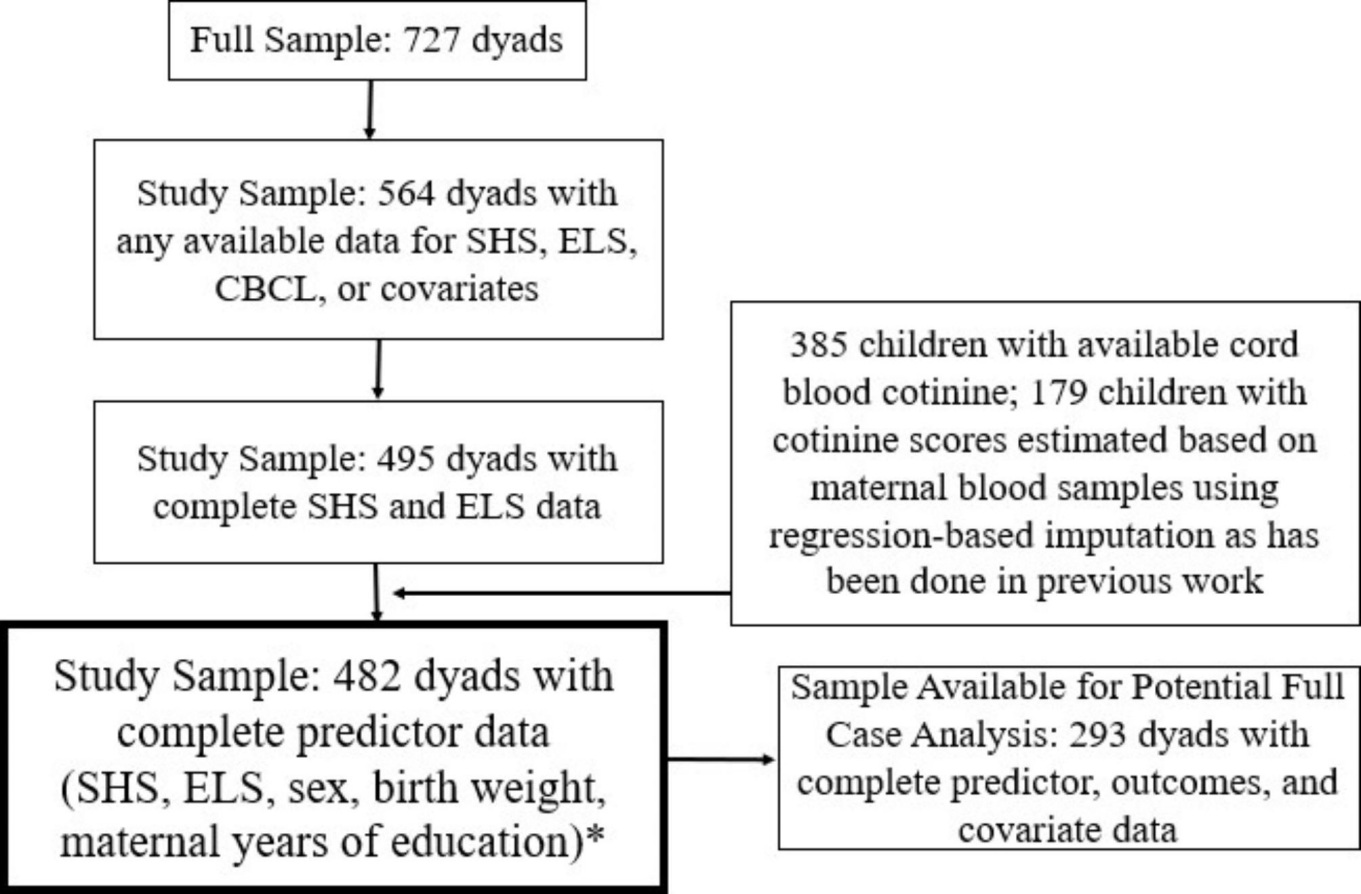
Flow Chart of Participant Inclusion. *Latent growth models described in results analyzed data from 482 participants with complete predictor data. WQS analyzed all available outcome data at each timepoint as described in Table 1

Detailed demographic and recruitment information regarding the *Columbia Center for Children’s Environmental Health (CCCEH)* Mothers and Newborns prospective birth cohort have been previously published [49]. To briefly summarize, Black and Latinx women residing in Washington Heights, Harlem, or the South Bronx in New York City (NYC) were recruited between 1998 and 2006 through local prenatal care clinics. Recruited women were non-users of tobacco products or illicit drugs, between the ages of 18 and 35 years, and free of diabetes, hypertension, or known HIV, and had initiated prenatal care by the 20th week of pregnancy. The full cohort included data from 727 mother-child dyads. Of the 727 dyads enrolled in the Mothers and Newborns cohort, 564 had available data for all predictors of interest (i.e., SHS, ELS, sex, maternal years of education, and birthweight) and so were included in the current analyses (Fig. 1). This study was approved by the Institutional Review Board of Columbia University, and mothers provided informed consent for themselves and their children at every study visit. Children provided assent beginning at age 7.

**Table 1:** Demographic characteristics of participants at each study visit

	Preschool	Age 5	Age 7	Age 9	Age 11	Total	Full Case Analysis
N	505	513	479	445	362	564	299
Mean age (SD)	3.25 (0.99)	4.43 (0.50)	6.52 (0.51)	8.52 (0.51)	10.56 (0.58)	-	-
Sex (% male)	238 (47.13)	244 (47.56)	226 (47.18)	207 (46.52)	165 (45.58)	272 (48)	130 (43.48)
Maternal years of education at prenatal visit (SD)	11.89 (2.18)	11.9 (2.16)	11.87 (2.14)	11.89 (2.13)	11.96 (1.95)	11.89 (2.16)	11.94 (1.83)
Child birthweight in grams (SD)	3376 (469.44)	3379 (469.17)	3388 (479.98)	3371 (481.84)	3398 (481.07)	3371 (474.10)	3371 (477.99)
Early life stress composite score	0.30 (0.12)	0.30 (0.12)	0.30 (0.12)	0.30 (0.12)	0.30 (0.13)	0.30 (0.12)	0.30 (0.12)
Cotinine^a^	-2.82 (1.65)	-2.82 (1.67)	-2.86 (1.60)	-2.84 (1.57)	-2.71 (1.61)	-2.81 (1.69)	-2.70 (1.66)
Latinx (%)	315 (62)	317 (62)	296 (62)	272 (61)	227 (63)	355 (63)	183 (61)
Black (%)	190 (38)	196 (38)	183 (38)	173 (39)	135 (37)	209 (37)	116 (39)

^a^. Z-scaled natural logarithm of prenatal cotinine exposure measured in maternal blood or cord blood samples taken at birth

### Study Timeline

Longitudinal study visits began in the third trimester and occurred approximately every two years thereafter for each child in the cohort. Prenatal exposure to SHS was measured by cotinine in either maternal blood or cord blood samples taken at birth [50]. The current study leverages behavioral data collected during four visits: preschool age (age range: 22–68 months; mean age = 42.85 months), age 5 (age range: 4–6 years; mean age = 4.43 years), age 7 (age range: 6–8 years; mean age = 6.52 years), age 9 (age range: 8–10 years; mean age = 8.52 years), and age 11 (age range: 10–13 years; mean age = 10.56 years). Measures of children’s socioemotional functioning were collected during children’s preschool (3–5 years), age 7, age 9, and age 11 year visits; ELS was measured at a separate children’s age 5 year study visit.

### Measures

#### Children’s Socioemotional Functioning

Children’s behavior problems were measured via parent report on the Child Behavior Checklist ( [51]; CBCL). We used the CBCL 1.5-5, a 100-item measure for children ages 1.5 to 5 years old which includes Emotionally Reactive, Anxious/Depressed, Somatic Complaints, Withdrawn, Sleep Problems, Attention Problems, and Aggressive Behavior subscales and the CBCL 6–18, a 113-item scale which includes Anxious/Depressed, Withdrawn/Depressed, Somatic Complaints, Social Problems, Thought problems, Attention Problems, Rule-Breaking Behavior, Aggressive Behavior subscales. The internalizing problems summary composite score for the CBCL 1.5-5 encompasses responses from the Emotionally Reactive, Anxious/Depressed, Somatic Complaints, and Withdrawn subscales. The internalizing problems summary composite score for CBCL 6–18 encompasses responses from Anxious/Depressed, Withdrawn/Depressed, and Somatic Complaints syndrome scales. Mothers were administered the CBCL age 1–5 at the child’s preschool visit and the CBCL 6–18 for children’s age seven, nine, and eleven visits.

#### Maternal stress during children’s early life

At child age 5, mothers completed a structured interview with a trained research assistant assessing demographics and six domains of early life stress exposure. These measures assess maternal experiences of stress during the child’s early life; as such, we refer to them as early life stress (ELS) because they reflect the shared experience of stress between mother and child. Items were aggregated across domains of stress exposure to create a single composite score, as has been done in previous work [52]. Items included in the composite score were extracted from several published scales examining past year material hardship [53], past month maternal perceived stress [54], intimate partner violence experienced during the child’s lifetime [55], lack of current social support [56, 57], current neighborhood quality [58–60], and past year nonspecific maternal distress or demoralization (Supplementary Methods; [61]). Independently, these scales demonstrate good reliability and psychometric properties [54, 57, 62–64]. All item responses were rescaled to 0–1 for the current analysis with higher scores indicating more stress exposure. Responses were averaged within each of the six domains, and then averaged across domains to create the composite score (range = 0–1, Mean = 0.30, Supplementary Fig. 1). This age 5 ELS composite score is used in all primary analyses described below. Because some CBCL preschool measures were taken at child age 3 (before the age 5 ELS measures were acquired) we generated an abbreviated stress composite consisting of measures of material hardship, maternal stress, and maternal demoralization, as these were the only measures available at age 3. We use this age 3 abbreviated ELS score to examine the correlation between age 3 and age 5 ELS and justify our use of age 5 ELS, even though some children were seen for CBCL slightly prior to when ELS was measured at age 5 (Supplementary Results; Supplementary Fig. 2).

#### Secondhand Tobacco smoke exposure

Cord blood samples were used whenever available (N = 500). If a cord blood sample was not available then SHS exposure was estimated using maternal blood samples (N = 197). Reported maternal smoking during pregnancy was exclusionary for enrollment in the cohort. Children born with cotinine concentrations in their cord blood above 25 ng/ml (N = 6) were excluded from this analysis because they were likely exposed to active smoking during gestation [50]. Cotinine values were positively skewed and so the natural logarithm of prenatal cotinine was z-scaled before use in the models described below.

### Analytic Plan

We estimated a latent growth model of children’s internalizing symptoms assessed from preschool to age eleven years-old using the growth function of the lavaan package in R studio version 4.1.1 [65, 66]. Of note, latent growth curve models are distinct from latent class models and use the continuous effects of all predictors on the continuous, data-derived outcomes, namely the intercept and slope scores. Intervals between slope loadings were scaled to reflect the intervals (i.e., years) between study visits. Full information maximum likelihood estimation was used to address missing CBCL data. Model fit was assessed using root mean square error of approximation (RMSEA), comparative fit index (CFI), and the standardized root mean square residual (SRMR), as these are recommended for smaller sample sizes (N < 500 [67, 68]). The RMSEA should be close to zero with a significance value > 0.05 [69, 70], CFI > 0.90 [71, 72], SRMR < 0.08 [73]).

Prenatal cotinine, early life stress, and their interaction were included in latent growth models to examine our hypothesis that the SHS*ELS interaction would be associated with increases in children’s internalizing symptoms and which would remain present over time. To control for potential confounding, we included as covariates the following variables which are known to be associated with SHS and ELS exposure: birthweight [74], child sex at birth [75, 76], and maternal years of education (e.g., [77]) measured at the prenatal visit in all analyses. Cotinine, ELS, birth weight, and years of education were z-scaled. CBCL internalizing T-scores were standardized using a grand mean and standard deviation derived by pooling across all time points. The SHS*ELS interaction term was calculated by multiplying the standardized SHS and ELS scores for each participant. Sensitivity analyses including maternal self-reported alcohol use were conducted (Supplementary Results, Table S4).

To explore effects of the individual components of the ELS composite while controlling for effects of SHS, weighted quantile sum (WQS) regression estimated associations between co-exposure to the six correlated measures of postnatal ELS and children’s internalizing scores at each time point, controlling for sex, birth weight, maternal years of education, and cotinine. Separate WQS regressions were conducted for each age point and so leveraged all available data from participants with CBCL outcome data at each age point. The WQS index was constructed by summing the ranked concentrations (quintiles) of each individuals’ exposures multiplied by the relative strength of each predictor variable’s association with their internalizing scores. Importantly, WQS can determine the overall influence of the multiple early life stressors and identify the contribution of each of the individual stressors to the overall impact on internalizing symptoms [78, 79]. A higher WQS index reflects higher exposures to stress related to the outcome, while a lower WQS index indicates either lower exposures, or that the WQS index is unrelated to the outcome. Estimating the WQS index was performed across 100 bootstrap ensembles [79], thereby minimizing vulnerability to collinearity among predictors, and resulting WQS indices were tested in a traditional linear framework, as: g(µ) = β0 + β1 WQS + z′φ. G(µ) reflects an identity link function, given the continuous nature of the outcomes, β0 reflects the model intercept, β1 indicates the association between the WQS index and the outcome, and z′ indicates a vector of covariates. All WQS models were tested using negative and positive constraints in order to examine the direction of the effect (either negative or positive) of the mixture components on the outcome. Sensitivity analyses were conducted to test the resolution of quantiling using tertiles and quartiles.

## Results

### Participants

Table 1 presents demographic information for the study sample. Mothers included in this study did not differ from those who did not have available data on age or maternal education. Black mothers were more likely than Latinx mothers to be missing maternal and/or cord blood samples (χ^2^ = 5.24, *p* = 0.02; Table S1). Across the entire sample, unscaled ELS composite scores ranged from 0.07 to 1.20 (mean = 0.4, SD = 0.18). Full case analysis included 293 participants with complete data across all predictors, outcomes, and covariates (Fig. 1). Children included in the full case analysis were more likely to be female (χ^2^ = 5.97, *p* = 0.01; Table S2).

### Combined SHS and ELS exposure are associated with higher internalizing symptoms in preschool and over time

The latent growth model indicated excellent model fit (RMSEA = 0.05, RMSEA p-value = 0.38, CFI = 0.96, SRMR = 0.03; details in Supplementary Results). Interaction effects were observed such that children with higher SHS*ELS had higher internalizing problems scores during the preschool visit (β = 1.17, SE = 0.53, *p* = 0.03) and a slower decrease in internalizing problems over time when compared with children with lower SHS*ELS scores (β=-0.16, SE = 0.06, *p* = 0.01). Significant main effects show a positive association between ELS and internalizing problems during the preschool visit (β = 0.22, SE = 0.06, *p* < 0.01) and a negative association between SHS and the slope of internalizing problems (β=-0.05, SE = 0.02, *p* = 0.04). No other significant findings were observed (Table 2).

**Table 2:** Latent Growth Curve Model Results

Variable	Intercept			Slope			
	Coefficient	z-value	p-value		Coefficient	z-value	p-value
Sex	0.07	0.58	0.56		-0.001	-0.07	0.95
Birth Weight	0.02	0.31	0.76		0.001	0.15	0.88
Years of Education	-0.05	-1.63	0.10		0.003	0.81	0.42
SHS	0.00	0.004	0.99		-0.001	-0.16	0.87
ELS	0.22	3.60	0.00		0.01	0.79	0.43
Interaction (SHS X ELS)	0.14	2.19	0.03		-0.02	-2.49	0.01
Overall Model	0.673	1.94	0.05		-0.057	-1.35	0.18

Note: SHS = Secondhand Smoke Exposure; ELS = Early Life Stress

### Specific components of ELS are associated with children’s internalizing problems

WQS regression indicated that the weighted ELS index was positively associated with children’s internalizing scores at all age points. At age 5, every quintile-increase in the exposure index, a 0.27 (95% confidence interval: 0.16, 0.40) increase in children’s internalizing problems scores was detected (p < 0.001; Table 3). Maternal demoralization, intimate partner violence, and perceived stress particularly contributed to children’s internalizing scores at age 5 (weight > 45%, 19%, 19% respectively; Table 4). The contribution maternal perceived social support, neighborhood quality, and material hardship to this association was negligible (weights ≤ 14%). Negative constraints yielded no significant results.

**Table 3:** Weighted Quantile Sum Results

	Coefficient	t-value	p-value
Age 5			
WQS	0.27	5.40	> 0.001
Sex	-0.002	-0.02	0.98
Birth Weight	-0.08	-1.27	0.21
Years of Education	-0.05	-2.09	0.04
SHS	-0.04	-0.55	0.58
Age 7			
WQS	0.37	6.03	> 0.001
Sex	0.23	2.04	0.04
Birth Weight	0.08	1.33	0.19
Years of Education	-0.03	-1.03	0.31
SHS	-0.05	-0.79	0.43
Age 9			
WQS	0.17	2.66	0.008
Sex	0.03	0.26	0.80
Birth Weight	0.10	1.44	0.15
Years of Education	-0.02	-0.79	0.43
SHS	0.09	1.24	0.22
Age 11			
WQS	0.26	3.91	0.0001
Sex	-0.06	-0.41	0.68
Birth Weight	-0.04	-0.59	0.59
Years of Education	-0.05	-1.49	0.14
SHS	-0.08	-1.14	0.26

Note: WQS = weighted quantile sum mixture; SHS = Secondhand Smoke Exposure

**Table 4:** Weights of ELS Mixture Components at Each Age Point

Component	Tertile Weight	Quartile Weight	Quantile Weight
Age 5			
Maternal Demoralization	0.44*	0.40*	0.45*
Intimate Partner Violence	0.16	0.22*	0.19*
Maternal Perceived Stress	0.13	0.21*	0.19*
Maternal Perceived Social Support	0.27*	0.13	0.14
Neighborhood Quality	0.002	0.009	0.02
Material Hardship	0.01	0.01	0.02
Age 7			
Maternal Demoralization	0.29*	0.14	0.18*
Intimate Partner Violence	0.44*	0.51*	0.45*
Maternal Perceived Stress	0.09	0.15	0.18*
Maternal Perceived Social Support	0.07	0.06	0.004
Neighborhood Quality	0.03	0.05	0.10
Material Hardship	0.09	0.09	0.09
Age 9			
Maternal Demoralization	0.31*	0.34*	0.18*
Intimate Partner Violence	0.34*	0.32*	0.45*
Maternal Perceived Stress	0.12	0.15	0.18*
Maternal Perceived Social Support	0.15	0.16	0.004
Neighborhood Quality	0.002	0.01	0.10
Material Hardship	0.08	0.09	0.09
Age 11			
Maternal Demoralization	0.28*	0.31*	0.44*
Intimate Partner Violence	0.19*	0.18*	0.16
Maternal Perceived Stress	0.03	0.04	0.06
Maternal Perceived Social Support	0.22*	0.25*	0.008
Neighborhood Quality	0.03	0.01	0.02
Material Hardship	0.25*	0.21*	0.32*

*indicates significant contribution to the mixture model as defined by weight greater than the cutoff τ = 0.167, or the inverse of the number of elements in the mixture (7)

At age 7, every quintile-increase in the exposure index, a 0.44 (95% confidence interval: 0.32, 0.57) increase in children’s internalizing problems scores was detected (p < 0.001; Table 3). Intimate partner violence, maternal demoralization, and perceived stress particularly contributed to children’s internalizing scores at age 7 (weight > 45%, 18%, 18% respectively; Table 4). The contribution maternal perceived social support, neighborhood quality, and material hardship to this association was negligible (weights ≤ 10%). Negative constraints yielded no significant results.

At age 9, every quintile-increase in the exposure index, a 0.23 (95% confidence interval: 0.10, 0.37) increase in children’s internalizing problems scores was detected (p < 0.001; Table 3). Maternal demoralization, intimate partner violence, and perceived stress particularly contributed to children’s internalizing scores at age 9 (weight > 32%, 31%, 27% respectively; Table 4). The contribution maternal perceived social support, neighborhood quality, and material hardship to this association was negligible (weights ≤ 6%). Negative constraints yielded no significant results.

For every quintile-increase in the ELS exposure index, children’s age 11 internalizing problems scores increased by 0.26 scaled T-score points (95% confidence interval: 0.12, 0.40; p < 0.001; Table 3), controlling for effects of SHS. Maternal demoralization and material hardship contributed significantly to children’s internalizing scores at age 11 (weight > 44%, 32% respectively; Table 4). The contributions of maternal social support, neighborhood quality, intimate partner violence, and maternal perceived stress were not significant (weights ≤ 16%). Models examining tertiles, quartiles, and outcomes at other ages yielded similar results (Table 4). Negative constraints yielded no significant results.

## Discussion

The current study examined the interacting effects of maternal stress during children’s early life and SHS on children’s internalizing symptoms and their development over time. Exposure to SHS interacted with maternal stress during children’s early life to result in higher internalizing symptoms during the preschool period. In addition, they interacted to result in slower decreases in symptoms across childhood. Children with the highest levels of both SHS and ELS failed to show the expected normative decreases in internalizing symptoms when ELS and SHS are not present. By examining this interaction, we clarify prior equivocal findings of associations between SHS and internalizing symptoms. Critically, maternal demoralization and material hardship, but not the four other domains of ELS, were significantly associated with children’s internalizing symptoms across childhood. Such findings are consistent with translational work showing that material hardship causes behaviors in rodents analogous to internalizing problems in humans [80]. Our findings underscore the importance of longitudinal studies to understand effects of exposure on children’s mental health outcomes. Moreover, our study points to the importance of modeling effects of multiple sources of neurotoxic exposures, including those in both the chemical and social environment. Identifying and intervening on modifiable factors such as prenatal SHS, maternal demoralization, and material hardship may reduce the prevalence of internalizing problems in youth.

### SHS exposure primes vulnerability to ELS for preschool internalizing symptoms

Exposure to SHS and ELS resulted in higher internalizing problems scores at in preschool, supporting our hypothesis that the interaction between SHS and ELS would be associated with more internalizing symptoms. We were not able to look at causal or directional effects of SHS and ELS measured concurrently. However, we observed a positive association between ELS and internalizing problems in preschool, suggesting prenatal SHS exposure might compound effects of stress. Such conjecture is supported by animal models showing that prenatal nicotine exposure alters central nervous system nicotinic acetylcholine receptors (nAChR; [81]) which are involved in the development and regulation of dopaminergic systems [80–83]. These dopaminergic systems play a critical role in processing threat [83–86] and are disrupted by ELS [86–89]. Thus, the interactive effects of SHS and ELS may operate through effects on the dopaminergic system. Future studies should examine these pathways to identify potential pharmacologic treatment targets in youth with pollution and stress-related internalizing disorders.

### Combined SHS and ELS exposure is Associated with slower decline in internalizing symptoms across childhood

Combined exposure to SHS and ELS was associated with a slower decrease in internalizing symptoms over time, consistent with our hypothesis that the interaction between SHS and ELS would disrupt normative patterns of reduced internalizing symptoms across childhood [45, 90]. Our findings align with prior studies showing that maternal stress is associated with children’s increased internalizing symptoms over development [44, 46, 47]. If the combined targeting of dopamine circuits by SHS and ELS reorganizes neural development, these effects may become magnified over time. The reorganization of dopamine-dependent circuitry in infancy and early childhood may result in a behavioral phenotype that is primed for increased sensitivity to threat and greater expression of internalizing symptoms. In typical development, children overcome these challenges and there is a subsequent reduction in internalizing symptoms over time [45, 91, 92]. However, ELS exacerbates the internalizing phenotype, resulting in altered fear processing [92], hyperresponsivity to threat [93], and changes in dopamine functioning [92, 94, 95], which ultimately leads to greater vulnerability and risk for persistent internalizing symptoms [96]. Future work should examine these neural pathways as a possible mechanism for the interactive effects of ELS and SHS. Genetic risk for internalizing symptomatology may also increase vulnerability to the interactive effects for SHS and ELS. Given that our study was performed in an epidemiological birth cohort unweighted for clinical symptoms, we are unable to examine this question. Future studies examining similar exposures in a sample weighted for anxiety or mood problems would allow for the examination of gene by environment interactions.

We did not observe downward sloping trajectories of internalizing problems during pre-adolescence as has been observed in prior work [44–47]. Notably, prior studies of internalizing symptom trajectories in community youth have examined majority white, middle class samples [44–47]. Our sample consists entirely of Black and Latinx dyads, with the majority of our sample falling at or below the poverty line. Given the common co-occurrence of multiple stressors facing this population [18], including elevated parental psychological distress, racism, and economic hardship, it is likely that these factors contributed to relative stability in the slope of youth’s internalizing symptoms. As described below, our data support this hypothesis, such that distinct components of adversity contributed to children’s internalizing symptoms at every age.

### Distinct components of ELS contribute to internalizing symptoms beyond Effects of prenatal SHS

Examining components of the ELS composite revealed that maternal demoralization and material hardship significantly contributed to internalizing problems at age eleven whereas the other four domains of stress did not. Maternal demoralization reflects mothers’ subjective feelings of incompetence and associated feelings of psychological distress [97] that have been linked to risk for maternal depression [98, 99], which in turn has been linked to poor child emotional outcomes [99–103]. Material hardship – the inability to meet basic needs – has been directly linked with children’s risk for internalizing problems [104, 105]. In humans, material hardship increases risk for postpartum depression [105–108]. In rodent models, postpartum material hardship induces depression like behaviors in the dam, which in turn leads to disrupted maternal care-giving behaviors and altered dopamine functioning in offspring [80]. The combined targeting of dopaminergic systems by material hardship and maternal demoralization may serve as an etiologic mechanism for internalizing symptoms in offspring. Future translational research should examine the biological mechanism by which maternal demoralization and material hardship influence internalizing symptoms. Interventions addressing material hardship in young families, such as food stamps, unconditional cash transfer, and earned income tax credits, can improve health outcomes for both mothers and children [108–112]. Additionally, though understudied, interventions addressing maternal psychological distress show promising positive effects on parenting [112–116], which in turn has been linked to more positive health outcomes in youth [113, 117].

### Limitations

Our study is not without limitations. First, our cohort is not weighted for clinical outcomes, limiting our ability to examine trajectories of internalizing symptoms in both clinical and nonclinical populations. Future studies should examine these trajectories in a cohort weighted for clinical symptoms in order to assess these effects. Next, maternal demoralization is a proxy for maternal psychological distress, including maternal anxiety and depression; however, our study does not contain a clinical measure of maternal anxiety or depression symptoms. Future studies should include a measure of maternal psychopathology. Additionally, our stress composite score was measured during children’s age 5 study visit; whereas, our first measure of children’s internalizing symptoms occurred during their preschool (age 3–5) visit. Future studies should measure ELS prior to children’s first visit to understand the temporally predictive effects of these associations. Finally, data missingness may limit generalizability. Primary reasons for data missingness include inability to participate in a particular study visit, participants’ moving out of state, or loss of contact.

## Conclusions

Our study shows for the first time that combined exposure to prenatal SHS and maternal stress during children’s early life increases children’s internalizing symptoms in early childhood and that these effects persist across middle childhood. Clinically, our findings indicate that maternal demoralization and material hardship are important targets for personalized prevention and intervention to reduce the development of children’s internalizing problems. The study has a number of strengths including the relatively large prospective longitudinal birth cohort design that includes individuals who have historically been excluded from developmental research, as well as using a biomarker of exposure. Importantly, our study helps to clarify previous equivocal findings linking internalizing symptoms and SHS exposure by examining the interactive effects of SHS and ELS. It is possible that these interactive effects offer one potential mechanism through which prenatal SHS exposure may prime vulnerability to ELS and increase risk for psychopathology. Further these findings point to a pathway through mental health inequities may arise in Black youth, whose mothers are differentially exposed to SHS. Better understanding of the underlying mechanism of action of these profiles is necessary and may help address the current public health crisis in adolescent mental health [118, 119].

### Electronic supplementary material

Below is the link to the electronic supplementary material.

Supplementary Material 1

## Data Availability

This study leveraged data from the CCCEH Mothers and Newborns prospective, longitudinal birth cohort. Data is available upon written request.

## Abbreviations

SHS: Secondhand tobacco smoke (measured prenatally)
ELS: Maternal Stress During Children’s Early Life
CBCL: Child Behavior Checklist
CCCEH: Columbia Center for Children’s Environmental Health
WQS: Weighted quantile sum
