# Supplementary material for: Early life stress, prenatal secondhand smoke exposure, and the development of internalizing symptoms across childhood
Source: Environ Health. 2023 Aug 25;22:58. doi: 10.1186/s12940-023-01012-8 (PMC10463722; doi:10.1186/s12940-023-01012-8)
Supplement: Supplementary file 1 — Supplementary Material 1 [file 12940_2023_1012_MOESM1_ESM.docx]

**Supplementary Methods**

**Early Life Stress (ELS) Composite Score**

The ELS composite score was created by aggregating data from several published scales conducted during the standard CCCEH maternal interview. Published scales examine 6 measures of proximal poverty and ELS including 8 items examining material hardship, 14 items examining maternal perceived stress, 12 items examining mothers’ experience of intimate partner violence, 8 items examining maternal perceived social support, 32 items examining neighborhood quality, and 27 items examining maternal non-specific psychological distress or demoralization. All item responses were rescaled 0-1, with higher scores indicating greater ELS, as has been done in previous work (1). Reponses were averaged within each variable and then across all variables to create a single composite score. The composite score was used as an effect modifier in primary analyses. Follow up WQS regression analyses leveraged scores in each of the 6 areas as a mixture of ELS.

The material hardship portion of the interview assessed parent’s level of unmet basic needs in the areas of food, housing, and clothing, e.g., “In the past year has there been a time when you: 1. couldn't afford to buy food?; 2. couldn't afford a place to stay?; 3.couldn't afford gas/electricity?; or 4. couldn't afford clothing?”. Items examining maternal perceived stress were adapted from the Perceived Stress Scale (2) to assess mothers’ current stress level and perceived ability to cope with that stress. Intimate partner violence was examined using items adapted from the short from of the revised Conflict Tactics Scales (3), a widely used measure of intimate partner violence in epidemiological cohort studies. This scale measures both physical and psychological violence, including items such as “Caused a serious injury due to a fight you had” or “Yelled and screamed at you.” Perceived social support was measured using items adapted from Cohen’s interpersonal support evaluation list (4) and examined tangible, emotional, and informational support. Neighborhood quality was examined using items adapted from existing measures of neighborhood quality to assess social order/disorder (0-4 Likert scale; e.g., “Homeless/unemployed people are hanging around” ), safety and violence (0-4 Likert scale; e.g., “Letting children go outside during the day/night”), and social cohesion and neighborhood stability (yes/no agreement; e.g., “My neighborhood is a good place to live”). Maternal demoralization was measured using the Psychiatric Epidemiology Research Instrument–Demoralization (PERI-D; (5,6), a 27-item scale measuring eight composite domains of non-specific psychological distress (perceived physical health, sadness, poor self-esteem, dread, anxiety, confused thinking, hopelessness/helplessness, and psychophysiological symptoms).

**Scale Items**

8 items regarding material hardship

1. Think about where you live, the food you eat, and the things you can afford to do and buy. How do you feel about your overall living condition? Would you say?
2. In the last year, has there been a time when you and your family needed food but couldn't afford to buy it?
3. In the last year, has there been a time when you couldn't afford a place to stay, or when you couldn't pay the rent?
4. In the last year, has your gas or electricity been turned off because you couldn't afford to pay the bill?
5. In the last year, have you needed to buy any type of clothing for yourself or your family because you couldn't afford to pay for it?
6. In the last year, has there been a time when you or a member of your family needed medicine or medical care but didn't get the treatment because you couldn't afford it?
7. Do you currently receive Medicaid?
8. Do you currently receive any type of public assistance?

14 items regarding mother’s perceived stress

1. In the last month, how often have you felt that you were unable to control the important things in your life?
2. *In the last month, how often have you felt confident about your ability to handle personal problems?*
3. *In the last month, how often have you felt that things are going your way?*
4. In the last month, how often have you felt difficulties were piling up so high that you could not overcome them?
5. You have been upset because of something that happened unexpectedly.
6. You have felt nervous and “stressed”.
7. *You have dealt successfully with irritating life hassles.*
8. *You have felt that you were effectively coping with important changes that were occurring in your life.*
9. You have found that you could not cope with all the things you had to do.
10. *You have been able to control irritations in your life.*
11. *You have felt that you were on top of things.*
12. You have been angered because of things that happened that were outside of your control.
13. *You have found yourself thinking about things that you have to accomplish.*
14. *You have been able to control the way you spend your time.*

12 items regarding experience of intimate partner violence

Many of us have been forced into situations where we have felt scared or uncomfortable. I would like to ask you some questions regarding threats of physical or emotional harm. Since you became pregnant with your child, how often has your partner (Husband, Boyfriend, or any partner you have had since you became pregnant) done any of the following?

1. Threatened to hurt you in any way
2. Caused a serious injury due to a fight you had
3. Used other forms of force during a fight (kicked, pushed, shoved or slapped)
4. Insulted you or embarrassed you in front of others (friends/family/colleagues)
5. Sworn or cursed at you
6. Treated you like an inferior
7. Yelled and screamed at you
8. Monitored and accounted for your whereabouts
9. Been jealous or suspicious of your friends
10. Accused you of having an affair with someone else
11. Interfered in your relationship with other family members
12. Kept you from doing things to help yourself

8 items regarding social support

Now, I would like you to think about people you are close to. Think of the people you live with,

your family, and your friends.

1. *Do you think there is someone who feels very close to you?*
2. *Is there someone who you can lean on for support?*
3. *When you are happy, is there someone you can share it with... Someone who will feel happy simply because you are happy?*

I am going to mention several kinds of support most of us need at times. For each, please tell me whether you believe you could get this help if you need it. Do you know

1. *Someone that would take you to the doctor, if needed?*
2. *Someone that would loan you $100 if you needed it?*
3. *Someone that would help with daily chores if you were sick?*
4. *Someone that you could talk to about problems in your life?*
5. *Someone who would watch your children when needed?*

32 items about neighborhood quality

The next section contains questions about some troubles you may be experiencing that make your life difficult. We understand that these problems can be very distressing, and are asking these questions to better understand the things in life that make it hard for you to do your job as a mother. I am going to ask you questions about your neighborhood.

Please tell me how often these things happen in your neighborhood

1. *Trash is picked up by trash collector*
2. Litter or trash stays on the sidewalks or streets
3. *Houses and buildings are kept in good repair on the outside*
4. Graffiti on buildings and walls
5. Abandoned cars
6. *The neighborhood streets are busy with shoppers, mothers with strollers, and children playing*
7. Drug dealers make it difficult to feel safe on the street
8. Homeless or unemployed people are hanging around
9. There are vacant or boarded up buildings
10. Gang activity makes it difficult to feel safe
11. Houses and yards not kept up
12. Gunshots and crime make the neighborhood feel unsafe

How worried are you about the following things in your neighborhood?

1. Having property stolen
2. Walking alone during the day
3. Letting children do outside during the day
4. Letting children do outside at the night
5. Being robbed
6. Being murdered

I am going to read several statements about your neighborhood. As I read each one, please answer yes or no.

1. *My neighborhood is a good place to live*
2. *My neighborhood is a good place to raise children*
3. *The people moving into the neighborhood in the past year or so are good for the neighborhood*
4. I would like to move out of this neighborhood
5. *When the weather is nice, the people living on my street visit with one another outside*
6. *The people in my neighborhood visit one another in their homes*
7. *The people in my neighborhood loan things to one another*
8. *The people in my neighborhood make sure other’s homes are safe when someone is away*
9. *On Halloween, most of the children living here go trick-or-treating in my neighborhood*
10. People move in and out of my neighborhood a lot
11. There are some children in the neighborhood that I do not want my children to play with
12. Neighbors should mind their own business about other’s children
13. The people moving into the neighborhood lately are bad for the neighborhood
14. Do you ever avoid going outside for safety reasons?

27 items regarding nonspecific maternal distress or demoralization

Now we are going to ask you some questions about your feelings and your state of mind during the past year.

1. During the past year, how often have you felt you were bothered by all different kinds of ailments in different parts of your body…
2. During the past year, how often have you been bothered by feelings of sadness or depression – feeling blue…
3. In general, how satisfied have you been with yourself in the last year...
4. During the past year, how often have you had attacks of sudden fear or panic...
5. *During the past year, how often have you felt confident...*
6. During the past year, how often have you felt lonely...
7. During the past year, how often have you been bothered by feelings of restlessness
8. During the past year, how often have you felt useless...
9. During the past year, how often have you feared going crazy; losing your mind
10. During the past year, how often have you felt anxious...
11. During the past year, how often have you feared something terrible would happen to you...
12. During the past year, how often have you felt confused and had trouble thinking
13. During the past year, how often have you had trouble concentrating or keeping your mind on what you are doing
14. During the past year, how often have you felt that nothing turns out for you the way you want it to...
15. During the past year, how often have you felt completely hopeless about everything...
16. During the past year, how often have you felt completely helpless....
17. During the past year, how often have you had times when you couldn’t help wondering if anything was worthwhile anymore...
18. During the past year, how often have you been bothered by cold sweats...
19. During the past year, how often have you had trouble with headaches or pains in the head...
20. During the past year, how often has your appetite been poor....
21. *In general, if you had to compare yourself with the average woman your age, what grade would you give yourself for the past year....*

Are you the kind of person

1. *Who feels she has much to be proud of ...*
2. Who is the worrying type...
3. Who feels that she is a failure generally, in life...
4. When you have gotten angry in the last year, how often have you felt uncomfortable, like getting headaches, stomach pains, cold sweats and things like that...
5. During the past year, how often have you feared being left all alone or abandoned...
6. During the past year, how often have you been bothered by nervousness, being fidgety or tense.

**Supplementary Results**

Table S1. Demographic characteristics of included and excluded participants at the prenatal visit

|  | Study Participants | Excluded participants | F/ χ2 | p |
| --- | --- | --- | --- | --- |
| N | 564 | 163 | - | - |
| Child sex (% male) | 272 (48.40) | 78 (47.56) | 0.036 | 0.85 |
| Maternal age at prenatal visit | 24.67 (4.92) | 24.21 (5.00) | 1.14 | .29 |
| Maternal years of education at prenatal visit (SD) | 11.89 (2.16) | 11.66 (2.26) | 1.43 | .23 |
| Child birthweight in grams (SD) | 3370 (474.49) | 3372 (449.35) | 0.001 | 0.97 |
| Child gestational age in weeks (SD) | 39.21 (1.29) | 39.18 (1.73) | 0.04 | .85 |
| Premature (%) | 17 (3.62) | 9 (6.92) | 2.68 | .10 |
| Cotinine^a^ | -2.81 (1.69) | -2.66 (1.85) | 0.90 | .34 |
| Latinx (%) | 354 (63) | 119 (73) | 5.24 | .02 |
| Black (%) | 209 (37) | 45 (27) | 5.24 | .02 |

^a.^ Z-scaled natural logarithm of prenatal cotinine exposure measured in maternal blood or cord blood samples taken at birth.

Table S2. Demographic characteristics of all included participants and those in a complete case analysis sample at the prenatal visit

|  | Study Participants | Complete Case Analysis | F/ χ2 | p |
| --- | --- | --- | --- | --- |
| N | 564 | 299 | - | - |
| Child sex (% male) | 272 (48.40) | 130 (43.48) | 5.97 | 0.01 |
| Maternal age at prenatal visit | 24.67 (4.92) | 25.04 (5.10) | 3.37 | 0.07 |
| Maternal years of education at prenatal visit (SD) | 11.89 (2.16) | 11.94 (1.83) | 0.27 | 0.60 |
| Child birthweight in grams (SD) | 3370 (474.49) | 3371 (474.10) | 0 | 0.98 |
| Child gestational age in weeks (SD) | 39.21 (1.29) | 39.24 (1.28) | 0.19 | 0.66 |
| Premature (%) | 17 (3.62) | 9 (3.54) | 0.01 | 0.93 |
| Cotinine^a^ | -2.81 (1.69) | -2.70 (1.66) | 2.6 | 0.11 |
| Latinx (%) | 354 (63) | 183 (61) | 0.83 | 0.36 |
| Black (%) | 209 (37) | 116 (39) | 0.83 | 0.36 |

^a.^ Z-scaled natural logarithm of prenatal cotinine exposure measured in maternal blood or cord blood samples taken at birth.

*Girls were more likely to be included in the complete case analysis*

*Distribution of Maternal Stress Composite Scores*

*
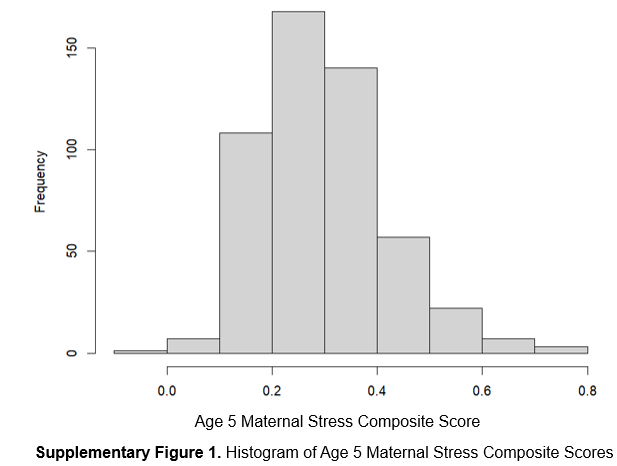
*

*Correlation Between Age 3 and Age 5 Stress Composite Scores*

Independent measures of ELS at age 3 and age 5 were moderately correlated. Correlations between composite scores of ELS at age 3 (consisting of all maternal demoralization, perceived stress, and material hardship; all *p*s<0.001) and at age 5 (consisting of all 6 maternal stress measures) were also significantly correlated (r(492) = .50, p <0.001).

*
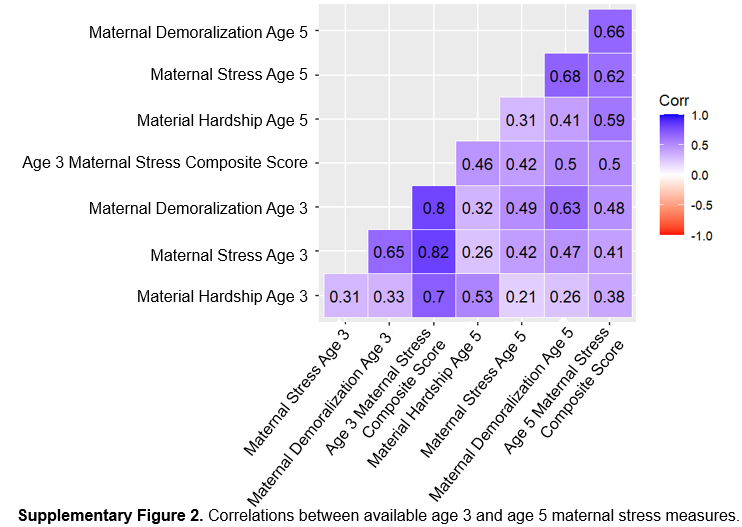
*

*Latent growth in Child Internalizing Symptoms*

Across the entire sample not accounting for exposures, an average internalizing problems score across participants was predicted at trend level (β=0.673; *p_intercept_*=0.053), indicating that the predicted preschool CBCL internalizing symptom scores was marginally different from 0. Similarly, across the entire sample not accounting for exposures, average internalizing problem scores did not change over time (β=-0.042; *p_slope_*=0.178). Finally, the covariance of the intercept and the slope was not significantly different from 0 (β=-0.021; *p*=0.206), indicating that child internalizing problems in preschool do not correlate with change in child internalizing problems over time when not accounting for exposures.

For completeness, we modeled a latent growth curve from age 7 to age 11 so that behaviors are only measured after the time of stress measurement. This model demonstrated similar results (Table S2). In sensitivity analyses, we also examined a latent growth curve model including mothers’ self-reported alcohol use during pregnancy; results were similar to results reported in the main text (Table S3). Finally, we examined a latent growth curve model including participant’s age at each study visit to control for differences in temporality between study visits across participants. Results were again similar (Table S4).

Table S3. Latent Growth Curve Model Results

| Variable | Intercept | | | Slope | | | |
| --- | --- | --- | --- | --- | --- | --- | --- |
|  | Coefficient | z-value | p-value | | Coefficient | z-value | p-value |
| Sex | 0.39 | 1.76 | 0.08 | | -0.04 | -1.41 | 0.16 |
| Birth Weight | 0.28 | 2.53 | 0.01 | | -0.03 | -2.29 | 0.02 |
| Years of Education | 0.01 | 0.20 | 0.84 | | -0.004 | -0.59 | 0.56 |
| SHS | 0.02 | 0.18 | 0.86 | | -0.004 | -0.31 | 0.76 |
| ELS | 0.31 | 2.63 | 0.01 | | -0.004 | -0.32 | 0.75 |
| Interaction (SHS X ELS) | 0.21 | 1.68 | 0.09 | | -0.03 | -1.93 | 0.05 |
| Overall Model | -0.20 | -0.29 | 0.77 | | 0.04 | 0.53 | 0.60 |

Note: SHS = Secondhand Smoke Exposure; ELS = Early Life Stress

Table S4. Latent Growth Model Results Including Self-Report Alcohol Use During Pregnancy

| Variable | Intercept | | | Slope | | | |
| --- | --- | --- | --- | --- | --- | --- | --- |
|  | Coefficient | z-value | p-value | | Coefficient | z-value | p-value |
| Alcohol Use During Pregnancy | 0.10 | 0.82 | 0.41 | | -0.007 | -0.49 | 0.62 |
| Sex | 0.04 | 0.32 | 0.75 | | 0.002 | 0.17 | 0.87 |
| Birth Weight | 0.03 | 0.48 | 0.63 | | 0.001 | 0.12 | 0.91 |
| Years of Education | -0.05 | -1.64 | 0.10 | | 0.003 | 0.83 | 0.41 |
| SHS | 0.02 | 0.30 | 0.76 | | -0.003 | -0.44 | 0.66 |
| ELS | 0.21 | 3.50 | 0.00 | | 0.006 | 0.82 | 0.41 |
| Interaction (SHS X ELS) | 0.13 | 2.01 | 0.04 | | -0.02 | -2.37 | 0.02 |
| Overall Model | 0.65 | 1.83 | 0.07 | | -0.06 | -1.30 | 0.19 |

Note: SHS = Secondhand Smoke Exposure; ELS = Early Life Stress

Table S5. Latent Growth Model Results Including Age at Each Time Point

| Variable | Intercept | | | Slope | | | |
| --- | --- | --- | --- | --- | --- | --- | --- |
|  | Coefficient | z-value | p-value | | Coefficient | z-value | p-value |
| Sex | 0.13 | 0.95 | 0.34 | | -0.01 | -0.49 | 0.62 |
| Birth Weight | 0.02 | 0.34 | 0.74 | | -.001 | -0.07 | 0.95 |
| Years of Education | -0.07 | -1.66 | 0.10 | | 0.004 | 0.84 | 0.40 |
| SHS | 0.05 | 0.66 | 0.51 | | -0.01 | -1.06 | 0.29 |
| ELS | 0.14 | 1.98 | 0.05 | | 0.01 | 1.76 | 0.08 |
| Interaction (SHS X ELS) | 0.12 | 1.62 | 0.10 | | -0.02 | -2.11 | 0.04 |
| Age at Preschool Visit | -0.10 | -1.28 | 0.20 | | 0.01 | 1.40 | 0.16 |
| Age at 5 Year Visit | 0.13 | 0.87 | 0.39 | | -0.01 | -0.80 | 0.42 |
| Age at 7 Year Visit | 0.10 | 0.67 | 0.50 | | 0.003 | 0.19 | 0.85 |
| Age at 9 Year Visit | -0.08 | -0.54 | 0.59 | | 0.01 | 0.72 | 0.47 |
| Age at 11 Year Visit | -0.08 | -0.69 | 0.49 | | 0.01 | 0.70 | 0.49 |
| Overall Model | 1.54 | 0.87 | 0.38 | | -0.27 | -1.35 | 0.18 |

Note: SHS = Secondhand Smoke Exposure; ELS = Early Life Stress

Supplementary References

1. Pagliaccio D, Herbstman JB, Perera F, Tang D, Goldsmith J, Peterson BS, et al. Prenatal exposure to polycyclic aromatic hydrocarbons modifies the effects of early life stress on attention and Thought Problems in late childhood. J Child Psychol Psychiatry. 2020 Nov;61(11):1253–65.

2. Cohen, Sheldon, Kamarck, T., and Mermelstein, R. Perceived stress scale. Journal of Health and Social Behavior [Internet]. 1983; Available from: https://www.northottawawellnessfoundation.org/wp-content/uploads/2018/04/PerceivedStressScale.pdf

3. Straus MA, Hamby SL, BONEY-McCOY SUE, Sugarman DB. The revised Conflict Tactics Scales (CTS2). J Fam Issues. 1996 May;17(3):283–316.

4. Cohen S, Mermelstein R, Kamarck T, Hoberman HM. Measuring the functional components of social support. In: Social Support: Theory, Research and Applications. Dordrecht: Springer Netherlands; 1985. p. 73–94.

5. Vernon SW, Roberts RE. Measuring nonspecific psychological distress and other dimensions of psychopathology. Further observations on the problem. Arch Gen Psychiatry. 1981 Nov;38(11):1239–47.

6. Dohrenwend BP, Shrout PE, Egri G, Mendelsohn FS. Nonspecific psychological distress and other dimensions of psychopathology. Measures for use in the general population. Arch Gen Psychiatry. 1980 Nov;37(11):1229–36.

7. Carrico C, Gennings C, Wheeler DC, Factor-Litvak P. Characterization of weighted quantile sum regression for highly correlated data in a risk analysis setting. J Agric Biol Environ Stat. 2015 Mar;20(1):100–20.
